# Tumor Compactness based on CT to predict prognosis after multimodal treatment for esophageal squamous cell carcinoma

**DOI:** 10.1038/s41598-019-46899-x

**Published:** 2019-07-19

**Authors:** Qifeng Wang, Bangrong Cao, Junqiang Chen, Chen Li, Lijun Tan, Wencheng Zhang, Jiahua Lv, Xiqing Li, Miyong Xiao, Yu Lin, Jinyi Lang, Tao Li, Zefen Xiao

**Affiliations:** 1 Department of Radiation Oncology, Sichuan Cancer Hospital & Institution, Sichuan Cancer Center, School of Medicine, University of Electronic Science and Technology of China, Radiation oncology Key Laboratory of Sichuan Province, Chengdu, 610041 China; 2 Department of Radiation Oncology, National Cancer Center/Cancer Hospital, Chinese Academy of Medical Sciences and Peking Union Medical College, Beijing, 100021 China; 3 Department of Radiation Oncology, Fujian Cancer Hospital & Fujian Medical University Cancer Hospital, Fuzhou, 350014 China

**Keywords:** Cancer imaging, Prognostic markers, Software

## Abstract

We aimed to establish a risk model using computed tomography-based compactness to predict overall survival (OS) and progression-free survival (PFS) after multimodal treatment for esophageal squamous cell carcinoma (ESCC). We extracted pre-treatment computed tomography-based tumor data (volume, surface area, and compactness) for 512 cases of ESCC that were treated at 3 centers. A risk model based on compactness was trained using Cox regression analyses of data from 83 cases, and then the model was validated using two independent cohorts (98 patients and 283 patients). The largest cohort (283 patients) was then evaluated using the risk model to predict response to radiotherapy with or without chemotherapy. In the three datasets, the pre-treatment compactness risk model provided good accuracy for predicting OS (P = 0.012, P = 0.022, and P = 0.003) and PFS (P < 0.001, P = 0.003, and P = 0.005). Patients in the low-risk group did not experience a significant OS benefit from concurrent chemoradiotherapy (P = 0.099). Furthermore, after preoperative concurrent chemoradiotherapy, the OS outcomes were similar among patients in the low-risk group who did and did not achieve a pathological complete response (P = 0.127). Tumor compactness was correlated with clinical T stage but was more accurate for predicting prognosis after treatment for ESCC, based on higher C-index values in all three datasets. The compactness-based risk model was effective for predicting OS and PFS after multimodal treatment for ESCC. Therefore, it may be useful for guiding personalized treatment.

## Introduction

In China, esophageal cancer was associated with a diagnosis rate of 477.9 cases/100,000 population and a mortality rate of 375/100,000 population during 2015^1,2^. The standard treatment for locally advanced esophageal squamous cell carcinoma (ESCC) is multimodal treatment including definitive chemotherapy and radiotherapy (CRT) or preoperative CRT^3–7^. However, it is important to predict the patient’s prognosis and response to multimodal treatment to enhance their management. The current esophageal treatment guidelines are mainly based on clinical or pathological staging, although the clinical stage cannot predict patients’ response to multimodal treatment^4,8–10^. This is because clinical staging is based on various examinations, including computed tomography (CT), esophageal ultrasonography, positron emission tomography–computed tomography (PET-CT)^11^, and magnetic resonance imaging (MRI)^12,13^. It is possible that various imaging-related parameters have prognostic value in this setting, such as CT-based or MR-based volume, however the volume can be affected by many reasons such as different oncologists or check equipment^13^. Therefore, tumor volume affected by multiple factors is hardly an effective prognostic predictor. Interestingly, CT-based compactness, which is calculated based on the primary tumor’s volume and surface area, is a prognostic factor in head, neck, and lung cancers^14–16^. Compactness is defined as a numerical quantity that can be calculated for three-dimensional objects as a function of the volume and surface area.However, to the best of our knowledge, no studies have examined the prognostic value of ESCC compactness or its correlations with treatment response, TNM stage, and radio-sensitivity. Therefore, the present study aimed to develop and validate a CT-based compactness risk model for predicting prognosis and the response of ESCC to multimodal treatment.

## Materials and Methods

### Study design

**Figure 1:**
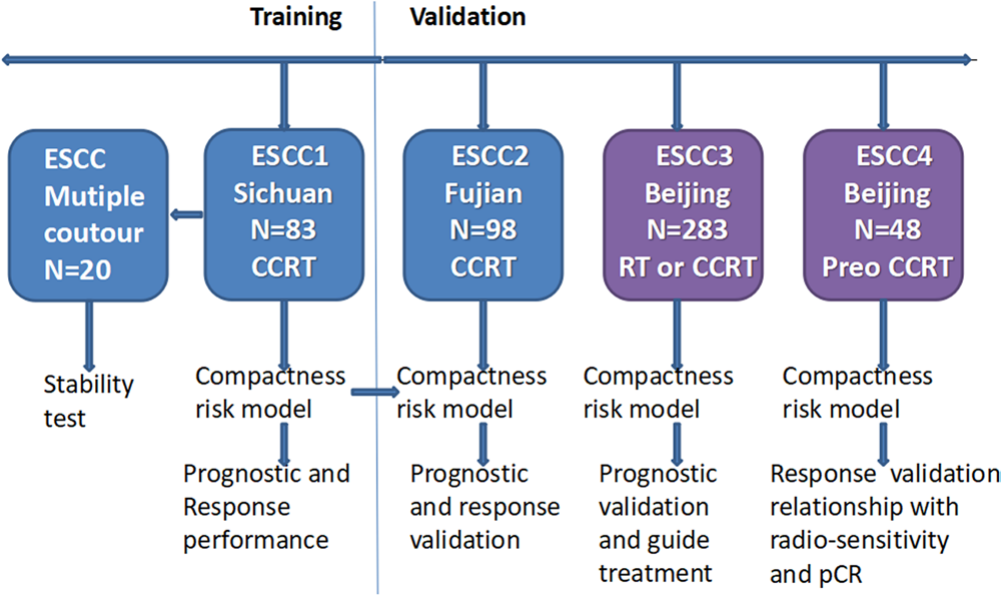
The definition of compactness was applied to the four datasets. One dataset used for training to determine the model’s value for predicting prognosis and treatment response. The other three datasets were used to validate the model and clarify the relationships between compactness, clinical TNM staging, and radio-sensitivity. CCRT: concurrent chemoradiotherapy, RT: radiotherapy, Preo: preoperative, pCR: pathological complete response.

The present study evaluated data from three separate cancer centers, and the institutional review boards of each center(National Cancer Center, Sichuan Cancer Center and Fujian Cancer Hospital) approved the study’s retrospective protocol. The risk model was created based on separate datasets from the participating centers, which included pre-treatment, imaging, treatment, and outcome data. Disease staging was performed according to the sixth edition (2002) of the AJCC staging manual^17^. The characteristics of the patients in the training and validation datasets are listed in Table 1. The treatment details are provided in the Supplementary Information. The study’s design is shown in Fig. 1.

**Table 1:** Characteristics of the patients in each dataset.

	ESCC1 training	ESCC2 validation	ESCC3 validation	ESCC4 validation
	N = 83 (%)	N = 98 (%)	N = 283 (%)	N = 48 (%)
Age (years)				
<65	55 (66.3)	57 (58.2)	139 (49.1)	41 (85.4)
≥65	28 (33.7)	41 (41.8)	144 (50.9)	7 (14.6)
Sex				
Male	64 (77.1)	73 (74.5)	238 (84.1)	41 (85.4)
Female	19 (22.9)	25 (25.5)	45 (15.9)	7 (14.6)
KPS				
90	32 (38.6)	38 (38.8)	66 (23.3)	30 (62.5)
80	49 (59)	58 (59.2)	180 (63.6)	18 (37.5)
70	2 (2.4)	2 (2)	37 (13.1)	0
Location				
Cervix	0	16 (16.3)	15 (5.3)	0
Upper	38 (45.8)	28 (28.6)	120 (42.4)	5 (10.4)
Middle	41 (49.4)	40 (40.8)	107 (37.8)	32 (66.7)
Lower	4 (4.8)	14 (14.3)	41 (14.5)	11 (22.9)
Length				
<5 cm	39 (47.0)	37 (37.8)	97 (34.3)	5 (10.4)
≥5 cm	44 (53.0)	61 (62.2)	186 (65.7)	43 (89.6)
Clinical T stage				
T1	0	0	9 (3.2)	0
T2	11 (13.3)	9 (9.2)	36 (12.7)	2 (4.2)
T3	50 (60.2)	51 (52)	104 (36.7)	20 (41.7)
T4	22 (26.5)	38 (38.8)	134 (47.3)	26 (54.2)
Clinical N stage				
N0	0	12 (12.2)	50 (17.7)	9 (18.8)
N1	83 (100)	86 (87.8)	233 (82.3)	39 (81.2)
Clinical M stage				
M0	83 (100)	68 (69.4)	207 (73.1)	48 (100)
M1A	0	23 (23.5)	31 (11)	0
M1B	0	7 (7.1)	45 (15.9)	0
Clinical TNM stage (6^th^ version)				
I	0	0	4 (1.4)	0
IIA	0	8 (8.2)	24 (8.5)	4 (8.3)
IIB	6 (7.2)	4 (4.1)	0	2 (4.2)
III	77 (92.8)	56 (57.1)	179 (63.3)	42 (87.5)
IVA	0	23 (23.5)	31 (11)	0
IVB	0	7 (7.1)	45 (15.9)	0
pCR				
Yes	NA	NA	NA	21 (43.8)
No	NA	NA	NA	27 (56.2)

KPS: Karnofsky Performance Score, pCR: pathological complete response.

### Datasets

The training dataset (ESCC1) included data from 83 patients who participated in a prospective randomized study (NCT01551589) that examined involved field irradiation and elective nodal irradiation for esophageal cancer^18^. The patients had undergone concurrent chemoradiotherapy (CCRT) for locally advanced ESCC at the Sichuan Cancer Center, and had available data regarding the CT simulation, gross tumor volume (GTV) delineations, clinical TNM stage (IIB–III), and survival outcomes. This dataset was used to assess the ability of tumor compactness to predict prognosis and treatment response, and to identify the optimal cut-off value for the risk model. In this dataset, 20 patients were randomly selected for multiple contour analysis by different oncologists.

The first validation dataset (ESCC2) consisted of 98 patients who underwent CCRT for ESCC at the Fujian Cancer Center. These patients also had available data regarding the CT simulation, GTV delineations, PET-CT findings, clinical TNM stage (IIB–IVB), and survival outcomes. This dataset was used to evaluate the ability of tumor compactness to predict prognosis and treatment response, as well as the relationships with TNM stage and lymph node metastasis.

The second validation dataset (ESCC3) consisted of 283 patients who were treated for ESCC (56 patients received CCRT and 227 patients received RT alone) at the National Cancer Center (Beijing). These patients also had available data regarding CT simulation, GTV delineations, ultrasonography findings, clinical TNM stage (I–IVB), and survival outcomes. This dataset was used to evaluate the ability of tumor compactness to predict prognosis and treatment response, as well as the relationships with clinical T stage and radio-sensitivity.

The third validation dataset (ESCC4) consisted of 48 patients who underwent preoperative CCRT at National Cancer Center. This dataset was used to evaluate the relationship between tumor compactness and radio-sensitivity.

### CT data acquisition and compactness calculation

All patients underwent radiotherapy (3D-CRT or IMRT) with or without chemotherapy. The CT data were downloaded from each center’s treatment planning system (step 1, Figure S1). Using Imaging Biomarker Explorer software (IBEX version 1.0β)^19^, the CT data were imported (step 2) and the GTV delineations were performed by three radiation oncologists in each center (steps 3 and 4). Esophageal stents, nasogastric tubes, intraluminal air, and oral contrast material were excluded from the GTV, and then descriptors were created for the GTV’s three-dimensional size and shape (step 5). Compactness is defined as a numerical quantity that can be calculated for three-dimensional objects as a function of the volume and surface area (step 6):

$${\rm{Compactness}}=({\rm{volume}})/[(\surd \pi )\times {({\rm{surface}}{\rm{area}})}^{2/3}].$$

### Data analysis

The analysis was divided into training and validation phases. For the training phase, we evaluated the prognostic values of compactness, volume, and surface area using Cox proportional hazards regression models, although compactness was selected because it had the best prognostic performance. In the training cohort, a dataset for 20 patients were separately delineated by four radiation oncologists(one vice chief doctor and three associated doctors), with delineation stability being evaluated using the Friedman test. The optimal compactness cut-off value for predicting survival was identified using x-tile software (version 3.6.1), and the patients were stratified according to their compactness values. For the validation phase, the compactness-based risk model was applied to three separate cohorts (ESCC2–4), and the model’s prognostic value was assessed using Kaplan-Meier curves and the log-rank test. Univariate Cox analyses were performed with several clinical variables, and multivariate Cox analysis was subsequently performed to determine whether the risk model was an independent prognostic factor. The associations between CT-based compactness and tumor length or TNM staging were also evaluated using Spearman’s rank correlation coefficient or the Mann-Whitney U test. The relative predictive values for tumor compactness, tumor length, and TNM staging were evaluated using each factor’s Harrell concordance index (C-index) value, with higher values indicating better ability to predict prognosis.

The ability of the model to predict treatment response was evaluated using the Beijing dataset (ESCC3) that included patients who underwent RT or CCRT. To further adjust for unbalanced factors, propensity score matching was performed to create comparable groups that underwent RT or CCRT. The propensity score for each patient was estimated using a logit model that included age, sex, tumor location, and clinical stage. Nearest neighbor matching (1:1) was then performed within a prespecified caliper width but without replacement. The survival benefits of CCRT and RT were compared using Kaplan-Meier curves and the log-rank test for the various compactness-based subgroups.

All statistical analyses were performed using IBM SPSS software (version 22.0; IBM Corp., Armonk, NT) and R software (version 3.2.0 for Microsoft Windows). Differences were considered statistically significant at two-tailed P-values of <0.05.

### Ethical approval

All procedures performed in studies involving human participants were in accordance with National Cancer Center, Sichuan Cancer Center and Fujian Cancer Hospital the ethical standards of the institutional and/or national research committee and with the 1964 Helsinki declaration and its later amendments or comparable ethical standards.

### Informed consent

Informed consent was obtained from all individual participants included in the study.

## Results

### Compactness is an independent prognostic factor for ESCC

**Figure 2:**
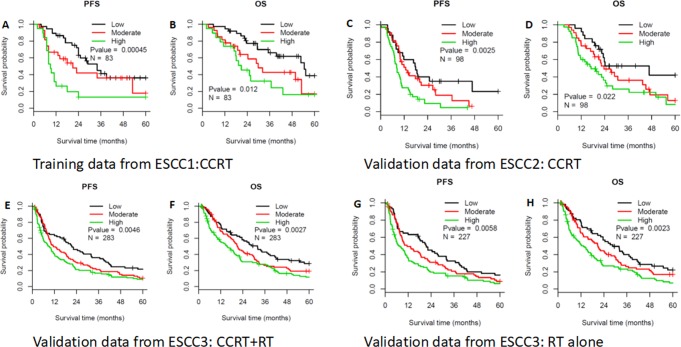
Prognostic value of the compactness-based risk model. Kaplan-Meier curves for overall survival (OS) and progression-free survival (PFS) are shown for the various datasets and compactness-based risk groupings (low, moderate, and high). P-values were calculated using the log-rank test. The OS and PFS results are presented for the training dataset (**A,B**) and the validation datasets from Fujian (**C,D**) and Beijing CCRT + RT alone (**E,F**) or Beijing RT alone (**G,H**).

In the training set, 103 patients with advanced ESCC (cT2–4N1M0) received CCRT during 2012–2016 as part of the NCT01551589 trial, although 20 patients were excluded from the present study based on M1 status (7 patients), age of >75 years (5 patients), and abnormal liver function (8 patients). Thus, 83 patients from that dataset were included in the present study and their characteristics are shown in Table 1. The median OS and PFS values for that group were 36.7 months and 24.0 months, respectively. Univariate analyses revealed that compactness (as continuous variable) predicted OS (HR = 2.74, 95% CI = 1.17–6.44, P < 0.02) and PFS (HR = 3.74, 95% CI = 1.57–8.88, P = 0.003). The x-tile software revealed two cut-off values for predicting compactness-based risk: low risk (<0.56), moderate risk (0.56–0.85), and high risk (>0.85) (Figure S2). The risk model significantly predicted PFS (Fig. 2A, P < 0.001) and OS (Fig. 2B, P = 0.012). The median OS times were 52.6 months in the low-risk group, 32.2 months in the moderate-risk group, and 20.8 months in the high-risk group. The median PFS times were 29.0 months in the low-risk group, 23.2 months in the moderate-risk group, and 9.0 months in the high-risk group. Multivariate Cox models indicated that the risk model based on compactness was able to independently predict OS and PFS in the training dataset (Table 2).

**Table 2:** Cox regression analyses of overall and progression-free survivals in the training data set.

Univariate analysis	Progression-free survival		Overall survival	
	HR (95% CI)	P-value	HR (95% CI)	P-value
Age				
<65 years	1		1	
≥65 years	0.6 (0.33–1.11)	0.102	0.55 (0.28–1.08)	0.081
Sex				
Male	1		1	
Female	0.85 (0.44–1.62)	0.617	0.77 (0.38–1.56)	0.464
KPS				
90	1		1	
80	0.97 (0.55–1.7)	0.903	1 (0.54–1.86)	0.989
70	5.21 (1.16–23.47)	0.032	3.38 (0.77–14.88)	0.107
Location				
Upper	1		1	
Middle	0.89 (0.5–1.57)	0.679	0.82 (0.44–1.52)	0.527
Lower	2.87 (0.98–8.37)	0.054	1.15 (0.34–3.9)	0.825
Length				
<5 cm	1		1	
≥5 cm	1.31 (0.75–2.26)	0.342	1.51 (0.83–2.76)	0.178
Clinical T stage				
T2	1		1	
T3	1.61 (0.67–3.88)	0.284	1.44 (0.55–3.76)	0.455
T4	1.89 (0.72–4.9)	0.194	1.81 (0.64–5.12)	0.262
TNM sixth edition				
IIb	1		1	
III	1.41 (0.51–3.93)	0.509	1.66 (0.51–5.38)	0.398
Response				
CR	1		1	
PR	1.08 (0.5–2.35)	0.837	1.46 (0.56–3.78)	0.435
SD	2.72 (1.07–6.92)	0.035	4.21 (1.42–12.46)	0.01
Compactness				
Low risk	1		1	
Moderate risk	1.61 (0.83–3.13)	0.156	2.02 (0.98–4.16)	0.056
High risk	3.69 (1.87–7.28)	<0.001	2.87 (1.38–5.97)	0.005
**Multivariate analysis**				
Age				
<65 years	1		1	
≥65 years	0.41 (0.19–0.87)	0.02	0.48 (0.21–1.07)	0.072
Sex				
Male	1		1	
Female	1.37 (0.65–2.91)	0.409	1.31 (0.57–3.01)	0.521
KPS				
90	1		1	
80	0.86 (0.45–1.62)	0.635	1.05 (0.53–2.07)	0.891
70	4.11 (0.82–20.61)	0.086	2.58 (0.5–13.46)	0.26
Location				
Upper	1		1	
Middle	1.17 (0.63–2.17)	0.615	1.1 (0.57–2.12)	0.781
Lower	5.21 (1.22–22.31)	0.026	0.95 (0.16–5.54)	0.952
Length				
<5 cm	1		1	
≥5 cm	1.49 (0.79–2.81)	0.215	1.54 (0.78–3.05)	0.212
TNM sixth edition				
IIb	1		1	
III	1.75 (0.56–5.51)	0.337	1.71 (0.47–6.28)	0.418
Response				
CR	1		1	
PR	1.23 (0.54–2.8)	0.615	1.48 (0.53–4.13)	0.458
SD	4.4 (1.56–12.43)	0.005	6.65 (2.06–21.49)	0.002
Compactness				
Low risk	1		1	
Moderate risk	2.48 (1.21–5.09)	0.013	2.73 (1.27–5.87)	0.01
High risk	3.37 (1.55–7.31)	0.002	3.13 (1.25–7.83)	0.015

HR: hazard ratio, CI: confidence interval, KPS: Karnofsky Performance Score, CR: complete response, PR: partial response, SD: stable disease.

The risk model was then validated using the Fujian dataset (98 patients in the ESCC2 dataset) and the Beijing dataset (283 patients in the ESCC3 dataset) (Fig. 2C–F), which revealed significant abilities to predict OS in the Fujian dataset (P = 0.022) and in the Beijing dataset (P = 0.003). The median OS times in the Fujian dataset were 46.7 months in the low-risk group, 23.6 months in the moderate-risk group, and 19.6 months in the high-risk group. The median OS times in the Beijing dataset were 31.9 months in the low-risk group, 20.8 months in the moderate-risk group, and 15.3 months in the high-risk group. The median PFS times in the Fujian data were 17.8 months in the low-risk group, 12.8 months in the moderate-risk group, and 8.4 months in the high-risk group (P = 0.003). The median PFS times in the Beijing dataset were 21.5 months in the low-risk group, 11.2 months in the moderate-risk group, and 8.6 months in the high-risk group (P = 0.005). Among 227 patients who only received RT in the Beijing dataset, the risk model was still significantly able to predict PFS (Fig. 2G, P = 0.006) and OS (Fig. 2H, P = 0.002).

Multivariate Cox models indicated that compactness was an independent prognostic factor for PFS in the two validations datasets (Table S1). Similarly, compactness was still able to significantly predict OS in Beijing (ESCC3) datasets, although it did not reach statistical significance in the Fujian dataset (ESCC2) (Table S1).

### Compactness is correlated with clinical T stage

**Figure 3:**
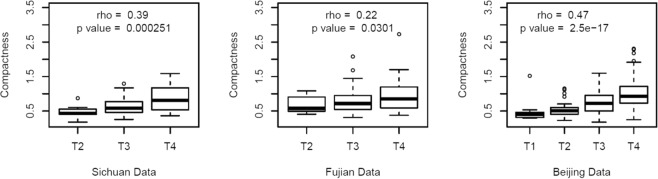
Compactness was significantly correlated with clinical T stage in the training dataset (P < 0.001), the Fujian dataset (P = 0.03), and the Beijing dataset (P < 0.001).

Compactness was significantly correlated with clinical T stage in the training dataset (Fig. 3A, P < 0.001), the Fujian dataset (Fig. 3B, P = 0.03), and the Beijing dataset (Fig. 3C, P < 0.001). Node metastasis is an important prognostic factor that can guide treatment for ESCC, although we detected a significant difference in the compactness values according to nodal status in the Beijing dataset (Figure S3, P = 0.015). In the Beijing dataset, the compactness-based risk model predicted prognosis among the 233 patients with N1 disease, which highlights the complementary nature of CT-based imaging and nodal status. There was no significant correlation of compactness with N stage in the Fujian dataset (P = 0.468). Furthermore, compactness was not significantly correlated with clinical M stage in the Fujian dataset (P = 0.152) or in the Beijing dataset (P = 0.598) (Figure S2).

### Compactness is better than clinical T stage for predicting ESCC prognosis

To compare the compactness-based model and clinical T stage, C-index values were calculated for each grouping’s ability to predict OS and PFS. In the training dataset (Table 3), the C-index values of compactness were 0.64 (95% CI: 0.55–0.73) for predicting OS and 0.66 (95% CI: 0.58–0.74) for predicting PFS. However, the C-index values of T stage were 0.58 for both OS and PFS. In the validation datasets (Fujian and Beijing), compactness also had higher C-index values that clinical T stage for predicting OS and PFS (Table 3). Compactness was also superior to the other TNM stages for both OS and PFS.

**Table 3:** Compactness and staging factors for predicting overall and progression-free survival.

	Progression-free survival				Overall survival			
	Variables	C-index	95% CI		Variables	C-index	95% CI	
Sichuan	Compactness	0.6612	0.5813	0.7411	Compactness	0.6410	0.5547	0.7273
training	T stage	0.5837	0.5067	0.6607	T stage	0.5849	0.5029	0.6669
	TNM	0.5277	0.4833	0.5720	TNM	0.5294	0.4824	0.5765
Fujian	Compactness	0.6040	0.5372	0.6709	Compactness	0.6073	0.5338	0.6808
validation	T stage	0.5089	0.4455	0.5724	T stage	0.5669	0.4966	0.6373
	N stage	0.5303	0.4885	0.5721	N stage	0.5462	0.4992	0.5932
	M stage	0.5192	0.4629	0.5755	M stage	0.5614	0.5015	0.6212
	TNM	0.5271	0.4644	0.5899	TNM	0.6015	0.5324	0.6706
Beijing	Compactness	0.5706	0.5326	0.6085	Compactness	0.5780	0.5382	0.6177
validation	T stage	0.5476	0.5102	0.5849	T stage	0.5521	0.5129	0.5913
	N stage	0.5245	0.4968	0.5522	N stage	0.5258	0.4966	0.5550
	M stage	0.5295	0.4991	0.5598	M stage	0.5199	0.4883	0.5515
	TNM	0.5446	0.5102	0.5789	TNM	0.5389	0.5029	0.5748

CI: confidence interval.

### Ability of the compactness-based risk model to guide treatment option

**Figure 4:**
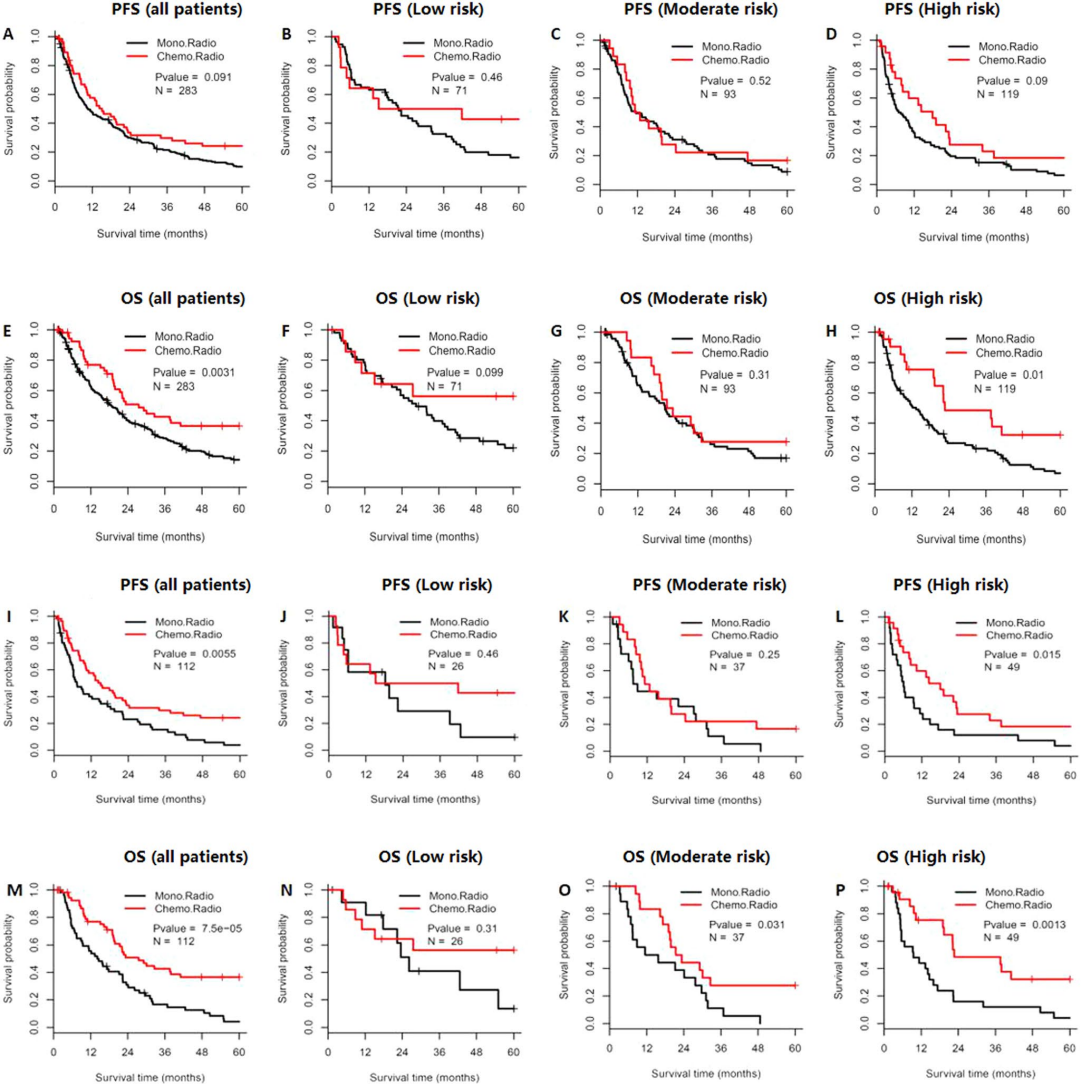
Survival benefit from concurrent chemoradiotherapy for esophageal squamous cell carcinoma according to the compactness-based risk model. The Kaplan-Meier curves for overall survival (OS) and progression-free survival (PFS) were compared for concurrent chemoradiotherapy (CCRT) and radiotherapy alone (RT) in the Beijing dataset (**A**–**H**). P-values were calculated using the log-rank test. The survival analyses were performed for all patients and patients in the low-risk, moderate-risk, and high-risk compactness subgroups. A subcohort from the Beijing dataset was generated using propensity score matching and subjected to the same analyses (**I**–**P**).

The Beijing datasets included 283 patients who were treated for ESCC (56 patients received CCRT and 227 patients received RT alone), with CCRT being associated with prolonged PFS (P = 0.091) and OS (P = 0.003) (Fig. 4A,E). In the high-risk compactness group, CCRT also provided prolonged PFS (P = 0.09) and OS (P = 0.01) (Fig. 4D,H). However, in the low-to-moderate risk compactness group, no significant differences in PFS (Fig. 4B,F) or OS (Fig. 4C,G) were observed between CCRT and RT alone. We also performed propensity score matching according to age, sex, KPS, and clinical TNM stage, which produced 56 matched pairs of patients who underwent CCRT or RT alone (Table S2). Among these patients, CCRT was associated with significantly prolonged PFS (P = 0.006) and OS (P < 0.001) (Fig. 4I,M). Similarly, in the high-risk compactness group, CCRT was associated with significantly prolonged PFS (P = 0.015) and OS (P = 0.001) (Fig. 4L,P). Among 10 randomly selected pairing results, 6 pairs revealed that the high-risk patients experienced a PFS benefit from CCRT, although no benefits were observed in the low-to-moderate compactness groups (Table S3). Moreover, all 10 pairing results indicated that the high-risk patients experienced an OS benefit from CCRT, although no benefits were observed in the low- or moderate-risk groups (Table S3).

### Compactness as a validated biomarker for treatment response

In the Sichuan dataset, the patients underwent RT (40 Gy in 20 fractions of 2 Gy), which provided a post-RT decrease in compactness for 65 of the 83 patients, with post-RT compactness of 0.56 significantly predicting both OS and PFS (both P < 0.001). In the Fujian dataset, post-treatment compactness (cut-off value: 0.56) also significantly predicted OS (P = 0.028) and PFS (P = 0.01). Moreover, among patients with moderate-to-high pre-treatment compactness, the post-treatment compactness also predicted OS (P = 0.052) and PFS (P = 0.002).

Among 48 patients in the Beijing dataset, pathological complete response (pCR) significantly predicted response to preoperative CCRT (OS: P = 0.02, PFS: P = 0.02). However, among patients without pCR, low-to-moderate compactness was associated with longer OS (P = 0.009) and PFS (P = 0.03) than in the high-risk group (Figure S4). Moreover, low- and moderate-risk patients with and without pCR had similar OS (P = 0.127) and PFS (P = 0.176) (Figure S3).

## Discussion

The present study revealed that CT-based compactness could predict OS and PFS among patients who received primary radiation-based treatment for ESCC. Furthermore, compactness was correlated with clinical T stage but was better for predicting prognosis in this setting. Therefore, tumor compactness reflects both the tumor burden and likelihood of treatment response. The clinical TNM stage is currently used to guide treatment, with staging performed based on findings from various imaging modalities (e.g., CT, MRI, PET-CT, and esophageal ultrasonography)^12^, although many hospitals lack sufficient equipment to accurately determine the clinical TNM stage. Thus, it would be useful to have a clear CT-based parameter for predicting TNM stage, the patient’s prognosis, and the likelihood of treatment response. Therefore, we evaluated tumor compactness in this setting, as a previous study has indicated that compactness was a prognostic factor for head, neck, and lung cancers^16^.

The burden of esophageal tumors may help predict treatment response based on the tumor’s length, thickness, volume, and surface area^13,20–22^. Furthermore, the present study revealed that compactness was a better prognostic marker than the tumor’s volume or surface area (Fig. S4), and compactness had better stability than volume and surface area in the multiple delineation analysis that involved different oncologists (Supplementary Data). Moreover, compactness was closely related to clinical T stage (a measure of tumor burden), although we detected center-specific differences in the correlations between compactness and T stage, which highlights the difficulty of standardizing clinical staging and subsequent treatment between centers. Otherwise, Clinical T staging only represents one dimension of tumor burden, and the increase of infiltration depth corresponds to the increase of T staging. However, tumor compactness represents tumor burden in multidimensional direction, including tumor volume and surface area. Therefore, tumor compactness is correlated with T stage, which can better represent tumor burden than T stage.

The RTOG8501 trial showed that CCRT significantly increased OS relative to RT alone^3^. However, 8% of the CCRT group experienced acute life-threatening toxic effects based on the RTOG acute morbidity scale and an additional 2% died as a direct consequence of treatment. In contrast, only 2% of patients who received RT alone experienced acute grade 4 toxic effects and there were no related deaths^3^. Thus, CCRT has significantly increased toxicity and cost, relative to RT alone, and remains a controversial treatment regimen especially for elderly patients with ESCC^23–25^, which highlights the importance of identifying patients who are not expected to benefit from CCRT. Interestingly, the present study revealed that patients with low-risk compactness did not benefit from CCRT, although further studies are needed to validate this relationship. Based on the results shown in Fig. 4B,F, it appears that patients who received CCRT had longer survival after 24 months, relative to patients who only received RT, and 14 patients who received CCRT had KPS scores of ≥80, while some patients who received RT alone had lower KPS scores (KPS 90: 29.8%, KPS 70: 14.0%; P = 0.05). Therefore, among patients in the low-risk group, KPS may help predict prolonged survival.

Among patients undergoing CCRT or preoperative CCRT, clinical TNM stage cannot accurately predict treatment response and prognosis^4,8,9,26^. In the Sichuan dataset (training) and the Fujian dataset (validation), the patients underwent CT examinations before and after treatment, with a post-treatment decrease in compactness (from ≥0.56 to <0.56) being associated with prolonged OS and PFS. Furthermore, after preoperative CCRT, the OS outcomes were similar among patients in the low-risk group who did and did not achieve a pCR (P = 0.127). Therefore, compactness may be a useful biomarker for predicting treatment response.

The present study used datasets from different centers and different radiation oncologists performed the GTV contouring, which is a potential limitation because clinical staging can vary between cancer centers. In this context, the Sichuan dataset involved prospectively enrolled patients with clinical stage IIB–III disease and a median OS of 36.3 months. In contrast, the Fujian and Beijing datasets were retrospectively obtained from patients with clinical stage I–IVb disease who underwent CCRT, RT alone, or preoperative CCRT. Thus, it is possible that differences in the prognostic value of compactness were related to the GTV contouring being performed by various oncologists at difference centers. Nevertheless, we did not detect any significant differences when we compared the contoured values from 4 oncologists for 20 patients. Another potential limitation is that the Sichuan dataset was smaller than the Fujian and Beijing datasets, with significantly lower proportions of patients with T4 and stage IV disease in the Sichuan dataset.

In conclusion, our findings indicate that tumor compactness can supplement the traditional evaluation of clinical T stage for ESCC. Furthermore, compactness based on volume and surface area independently predicted prognosis and treatment response in this setting.

## Supplementary information

supplementary information
