# Supplementary material for: Tumor Compactness based on CT to predict prognosis after multimodal treatment for esophageal squamous cell carcinoma
Source: Sci Rep. 2019 Jul 19;9:10497. doi: 10.1038/s41598-019-46899-x (PMC6642095; doi:10.1038/s41598-019-46899-x)
Supplement: Supplementary file 1 — supplementary information [file 41598_2019_46899_MOESM1_ESM.pdf]

Tumor Compactness based on CT to predict prognosis after multimodal treatment for esophageal squamous cell carcinoma

Running title: CT-based tumor compactness and ESCC prognosis

Qifeng Wang[1,*], MD, Bangrong Cao[1,*], PhD, Junqiang Chen[3,*] MD, Chen Li[2] MD, Lijun Tan[2], MD, Wencheng Zhang[2], MD, Jiahua Lv[1], MD, Xiqing Li[3], MD, Miyong Xiao[1], PhD, Yu Lin[3], MD, Jinyi Lang[1,#], MD, PhD,Tao Li[1,#], MD and Zefen Xiao[2,#], MD

[1] Department of Radiation Oncology, Sichuan Cancer Hospital & Institution, Sichuan Cancer Center, School of Medicine, University of Electronic Science and Technology of China, Radiation oncology Key Laboratory of Sichuan Province, Chengdu, 610041, China.

[2] Department of Radiation Oncology, National Cancer Center/Cancer Hospital, Chinese Academy of Medical Sciences and Peking Union Medical College, Beijing, 100021, China.

[3] Department of Radiation Oncology, Fujian Cancer Hospital & Fujian Medical University Cancer Hospital, Fuzhou, 350014, China.

* Three authors contribute equally to the work.

#**Corresponding author:**

Zefen Xiao, MD, Department of Radiation Oncology, National Cancer Center/Cancer Hospital, Chinese Academy of Medical Sciences and Peking Union Medical College, Beijing, China. E-mail: xiaozefen2013@163.com

Tao Li, MD, Department of Radiation Oncology, Sichuan Cancer Hospital& Institution, Sichuan Cancer Center, School of Medicine, University of Electronic Science and Technology of China, Chengdu, China. E-mail: litaoxmf@126.com

SUPPLEMENTARY METHODS AND DATA

Sichuan dataset

All patients underwent CT simulation in the supine position using a slice thickness of 3 mm. All simulator CT studies in our center were performed with a 16 detector row CT scanner (PHILIPS Healthcare) and the same clinical protocol: 120 kV, 180-280 mA with tube current modulation, a matrix of 512, a field of view of 500 mm, and 3mm reconstructed section thicknesses after intravenous injection of 60-120 ml of 370 mg/ml (1.5ml/Kg) iodinated contrast agent at 1.8-2 mL/sec.The gross tumor volume (GTV) was contoured according to previous clinical imaging results, such as esophageal radiography, enhanced computed tomography (CT), esophagoscopy, and positron emission tomography–computed tomography (PET-CT). The clinical target volume (CTV) was defined as the GTV plus a 2–3-cm margin in the craniocaudal direction and a 0.5-cm margin in the transverse plane, but not intruding on any anatomical barriers (e.g., blood vessels). The patients were randomized to receive either elective nodal irradiation (ENI) or involved field irradiation (IFI), with the CTVn of the ENI group including any involved lymph node regions and clinically uninvolved lymph node stations, based on the primary tumor's location. Lymph node station numbers 1/2/4/5/7, 2/4/5/7, and 4/5/7/16/17 were included for upper thoracic, middle thoracic, and lower thoracic ESCC in the ENI arm. The CTV of the IFI group included any clinically involved lymph node regions. The irradiation doses were 60–66 Gy to the target lesion and metastatic nodes and 50.4–54 Gy for the CTV.

Chemotherapy regimen and outcomes

The chemotherapy regimen included docetaxel (60–80 mg/m^2 on day 1) and cisplatin (25

mg/m^2 on days 1–3) administered in two 3-week cycles. The Sichuan dataset included 83 patients with stage IIB–III disease who were treated during 2012–2016. The median follow-up time was 40.1 months (range: 12.4–81.2 months), the median OS was 36.7 months, and the median PFS was 24.0 months. The 1-year, 3-year, and 5-year OS rates were 86.7%, 50.9%, and 28.3%, respectively. The 1-year, 3-yaer, and 5-year PFS rates were 66.2%, 32.6%, and 25.4%, respectively. There were no significant differences in OS and PFS between the ENI group (n=41) and the INI group (n=42).

Fujian dataset

All patients underwent CT simulation and three-dimensional images were reconstructed using the treatment planning system. The GTV was contoured according to previous clinical imaging results, such as esophageal radiography, enhanced CT, esophagoscopy, and PET-CT. The CTV was defined as the GTV plus a 2–3-cm margin in the craniocaudal direction and a 0.5-cm margin in the transverse plane, but not intruding on any anatomical barriers (e.g., blood vessels). For cervical and upper thoracic tumors, the CTVn included several lymphatic drainage areas (cervical, para-esophageal, and supraclavicular) and station numbers 2/3/4/7. For lower thoracic tumors, the CTVn included station 8 and several lymphatic drainage areas (peri-gastric and celiac axis). The median irradiation doses were 61.5 Gy (range: 50.0–67.7 Gy; 2.0–2.1 Gy per fraction) for the GTV and 54.0 Gy (range: 45–55.8 Gy; 1.8 Gy per fraction) for the CTV. Intensity-modulated radiotherapy was started 1 day after the first cycle of chemotherapy and administered 5 days/week for 5–6.6 weeks.

Chemotherapy regimen and outcomes

The chemotherapy regimen included docetaxel (135 mg/m^2 on day 1) and cisplatin (25 mg/m^2

on days 1–3) for two 3-week cycles. The median OS was 23.3 months and the 1-year, 3-year, and 5-year OS rates were 74.5%, 36.3%, and 18.2%, respectively. The median PFS was 10.4 months and the 1-year, 3-year, and 5-year PFS rates were 43.8%, 17.6%, and 8.2%, respectively.

Beijing dataset

The radiotherapy and chemotherapy plans were the same as for the Fujian dataset. The Beijing study included 330 patients during 2004–2014, although 47 patients were excluded from the present study because their pre-treatment primary CT images could not be retrieved. The median follow-up time was 42.3 months (range: 9.8–83.9 months), the median OS was 20.5 months, and the median PFS was 11.5 months. The 1-year, 3-year, and 5-year OS rates were 65.2%, 31.2%, and 18.7%, respectively. The 1-year, 3-year, and 5-year PFS rates were 49.3%, 23.6%, and 12.8%, respectively. Relative to patients who received RT, patients who received CCRT had better OS (18.8 months vs. 27.5 months, P=0.003) and better PFS (10.5 months vs. 15.1 months, P=0.092).

During that study, 49 patients underwent pretreatment using CCRT, although 5 patients were excluded from the present study because of loss to follow-up (n=1) or death before surgery (n=4). Among the remaining 44 patients, the median follow-up time was 42.3 months (range: 9.8–83.9 months), the median OS was not achieved, and the median PFS was 40.8 months. The 1-year, 3-year, and 5-year OS rates were 90.7%, 63.7%, and 55.8%, respectively. The 1-year, 3-year, and 5-year PFS rates were 79.1%, 61.3%, and 44.9%, respectively. Relative to patients without a pathological complete response, patients with a pathological complete

response had better median OS (26.5 months vs. not achieved, P=0.002) and better median PFS (15.3 months vs. 40.8 months, P=0.004).

CT image acquisition and compactness measurement

To evaluate the stability of the volume, surface area, and compactness measurements, 20 patients were randomly selected for GTV contouring by 4 different oncologists at two treatment centers. These data did not provide information regarding prognosis, although compactness generally provided greater stability and less delineation inaccuracy than tumor volume and surface area (compactness: Friedman chi-square = 4.56, P=0.207; surface area: Friedman chi-square = 22.14, P<0.001; volume: Friedman chi-square = 8.88, P=0.03).

Table S1: Cox regression analyses of progression-free and overall survivals in Fujian and Beijing dataset

	Progression-free survival		Overall survival	
	HR (95% CI)	P-value	HR (95% CI)	P-value
**Fujian data set**				
Age				
<65 years	1		1	
≥65 years	0.91 (0.56–1.5)	0.722	0.81 (0.47–1.41)	0.462
Sex				
Male	1		1	
Female	0.66 (0.37–1.21)	0.179	0.76 (0.38–1.49)	0.424
KPS				
90	1		1	
80	0.66 (0.4–1.11)	0.118	0.87 (0.52–1.46)	0.594
70	0.63 (0.11–3.62)	0.607	4.71 (0.73–30.46)	0.104
Location				
Cervical	1		1	
Upper	1.93 (0.72–5.18)	0.189	1.06 (0.36–3.06)	0.92
Middle	2.36 (0.92–6.04)	0.0746	1.46 (0.52–4.06)	0.469
Lower	3.53 (1.25–9.98)	0.0176	3.69 (1.2–11.39)	0.0229
Length				
<5 cm	1		1	
≥5 cm	1.18 (0.69–2.01)	0.542	1.44 (0.81–2.55)	0.215

TNM sixth edition				
IIa	1		1	
IIb	1.02 (0.2–5.06)	0.983	0.34 (0.03–3.73)	0.376
III	0.87 (0.34–2.18)	0.759	2.34 (0.59–9.2)	0.225
IVa	0.87 (0.32–2.4)	0.794	2.8 (0.66–11.8)	0.162
IVb	0.85 (0.25–2.94)	0.799	2.83 (0.58–13.9)	0.2
Compactness				
Low risk	1		1	
Moderate risk	1.46 (0.74–2.86)	0.275	1.82 (0.88–3.75)	0.104
High risk	2.05 (1–4.21)	0.0489	1.69 (0.78–3.67)	0.184
**Beijing data set**				
Age				
<65 years	1		1	
≥65 years	0.73 (0.55–0.96)	0.0238	0.79 (0.59–1.06)	0.117
Sex				
Male	1		1	
Female	0.9 (0.62–1.3)	0.58	0.73 (0.49–1.09)	0.128
KPS				
90	1		1	
80	0.97 (0.69–1.35)	0.839	0.92 (0.64–1.31)	0.629
70	1.43 (0.91–2.26)	0.118	1.35 (0.84–2.18)	0.209

Location				
Cervical	1		1	
Upper	0.86 (0.47–1.59)	0.638	0.59 (0.32–1.11)	0.103
Middle	0.8 (0.44–1.48)	0.485	0.54 (0.29–1.02)	0.0564
Lower	1.23 (0.62–2.43)	0.549	0.91 (0.45–1.8)	0.777
Length				
<5 cm	1		1	
≥5 cm	0.96 (0.69–1.33)	0.798	1.15 (0.81–1.64)	0.442
TNM sixth edition				
I	1		1	
IIa	2.3 (0.64–8.27)	0.203	2.87 (0.63–12.96)	0.171
III	3.14 (0.93–10.63)	0.066	4.09 (0.96–17.5)	0.0576
IVa	3.76 (1.07–13.19)	0.0387	5.38 (1.21–23.96)	0.0273
IVb	4.26 (1.22–14.93)	0.0235	4.91 (1.11–21.73)	0.0359
CCRT				
No	1		1	
Yes	0.59 (0.41–0.85)	0.00459	0.47 (0.31–0.69)	0.000153
Compactness				
Low risk	1		1	
Moderate risk	1.21 (0.82–1.79)	0.33	1.22 (0.8–1.86)	0.347
High risk	1.63 (1.09–2.44)	0.0168	1.64 (1.06–2.53)	0.0259

HR: hazard ratio, CI: confidence interval, KPS: Karnofsky Performance Score, CCRT: concurrent chemoradiotherapy.

Table S2. Propensity score matching for patients who received radiotherapy alone or concurrent chemoradiotherapy.

		After matching			Raw dataset		
		CCRT	RT	P-value	CCRT	RT	P-value
Sex				1			0.869
	Male	48 (85.7)	49 (87.5)		48 (85.7)	190 (83.7)	
	Female	8 (14.3)	7 (12.5)		8 (14.3)	37 (16.3)	
Age, years				0.496*			<0.001
	<60	39 (69.6)	35 (62.5)		39 (69.6)	64 (28.2)	
	60–70	15 (26.8)	16 (28.6)		15 (26.8)	71 (31.3)	
	>70	2 (3.6)	5 (8.9)		2 (3.6)	92 (40.5)	
KPS				0.744*			0.008
	90	21 (37.5)	17 (30.4)		21 (37.5)	45 (19.8)	
	80	32 (57.1)	35 (62.5)		32 (57.1)	148 (65.2)	
	70	3 (5.4)	4 (7.1)		3 (5.4)	34 (15)	
TNM stage				1*			0.147*
	I				0 (0)	4 (1.8)	
	IIA	1 (1.8)	2 (3.6)		1 (1.8)	23 (10.1)	
	III	40 (71.4)	39 (69.6)		40 (71.4)	139 (61.2)	
	IV	15 (26.8)	15 (26.8)		15 (26.8)	61 (26.9)	

* P-values were calculated using Fisher's exact test.

CCRT: concurrent chemoradiotherapy, RT: radiotherapy alone, KPS: Karnofsky Performance Score.

Table S3. Benefit from concurrent chemoradiotherapy for 10 randomly selected pairs of matched patients in each compactness risk group.

	CCRT vs. RT			
Pairing #	All patients	Low compactness	Moderate compactness	High compactness
**Progression-free survival**				
1	0.006	0.459	0.249	0.015
2	0.028	0.815	0.236	0.044
3	0.185	0.457	0.907	0.043
4	0.004	0.13	0.366	0.053
5	0.138	0.808	0.665	0.127
6	0.056	0.698	0.893	0.001
7	0.059	0.677	0.495	0.032
8	0.027	0.303	0.727	0.158
9	0.019	0.555	0.07	0.185
10	0.004	0.308	0.28	0.032
**Overall survival**				
1	<0.001	0.313	0.031	0.001
2	<0.001	0.677	0.044	0.004
3	0.01	0.42	0.346	0.001
4	<0.001	0.245	0.065	0.004
5	0.005	0.963	0.202	0.008
6	0.008	0.702	0.404	<0.001
7	0.001	0.383	0.11	0.001
8	<0.001	0.123	0.252	0.011
9	<0.001	0.646	0.012	0.01
10	<0.001	0.167	0.048	0.001

The table shows the log-rank p-values for the comparison of CCRT vs. RT. Among the 10 randomly selected pairs, patients in the high-risk group experienced a PFS benefit from CCRT in 6 groupings, although none of the patients in the low- and moderate-risk groups experienced a PFS benefit. In addition, 10 patients in the high-risk group experienced an OS benefit from CCRT, while none of the patients in the low- and moderate- risk groups experienced an OS benefit.

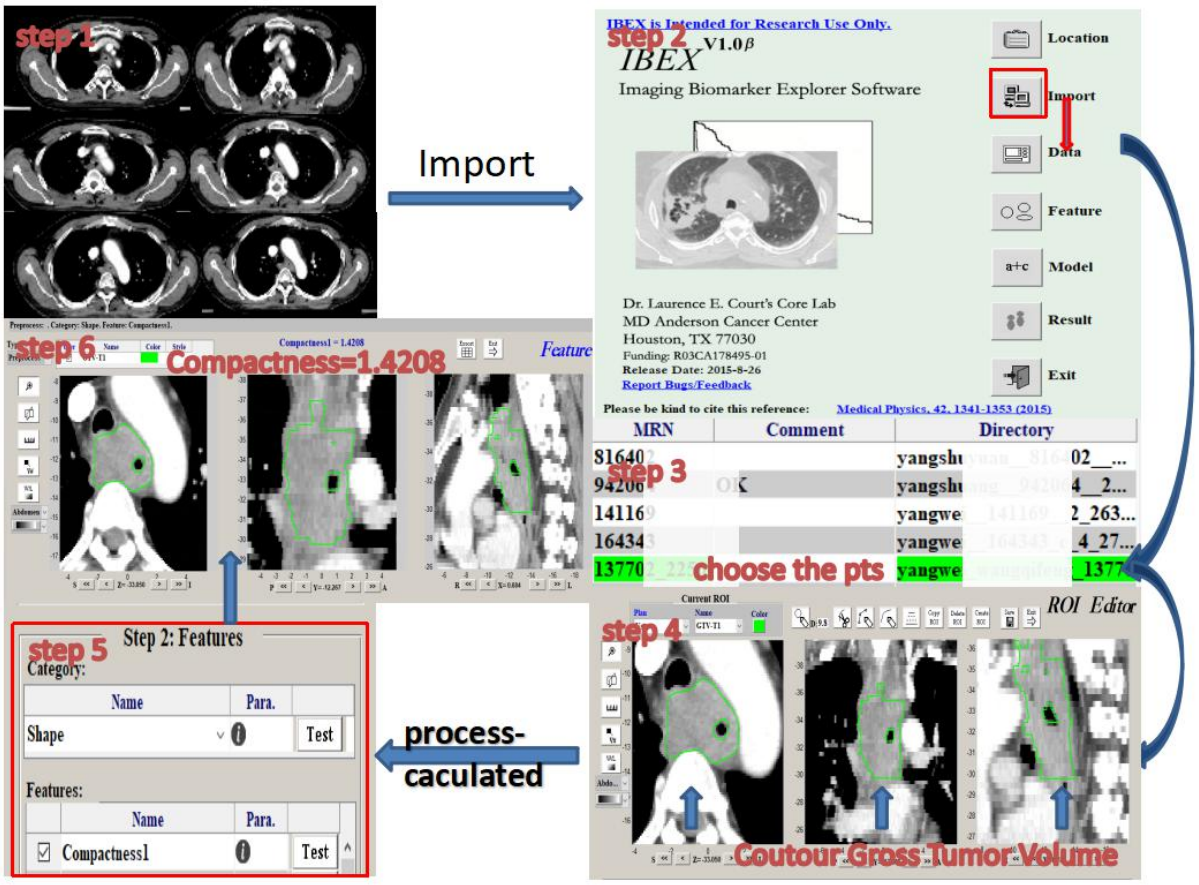

Figure S1: Measuring compactness based on computed tomography images [1-2].

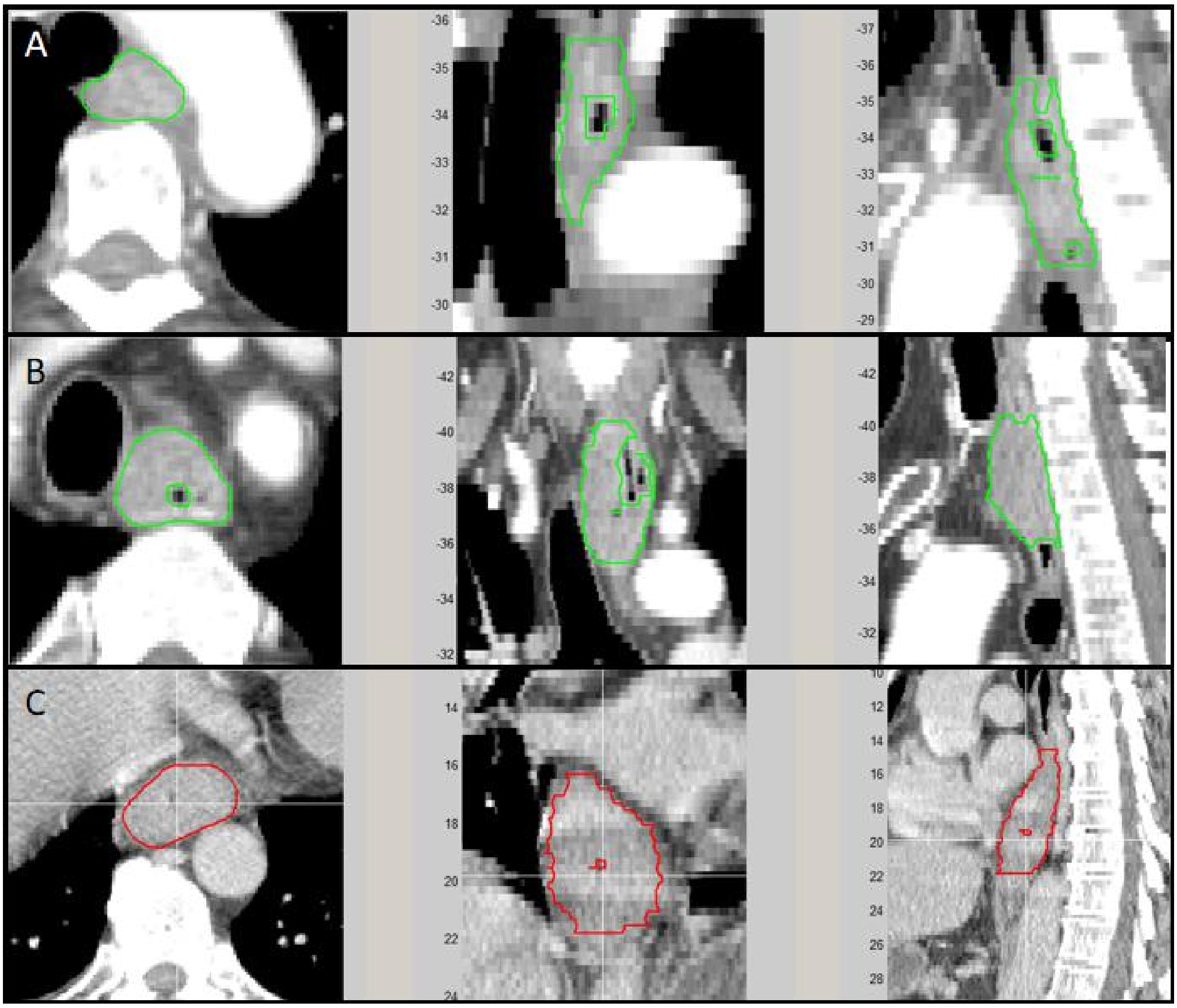

Figure S2： CT image examples of low risk(patients A), medium risk(patient B) , and high risk (patient C) for tumor compactness

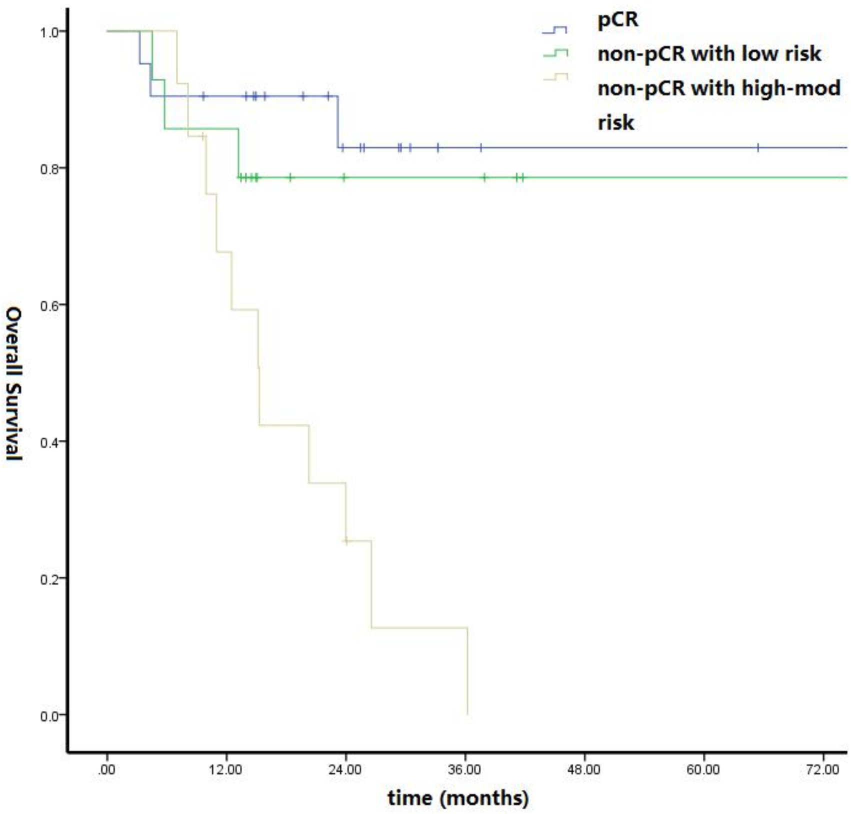

Figure S3: Among patients who did not achieve a pathological complete response, low-to-moderate compactness-based risk was associated with prolonged OS (P=0.009) relative to the high-risk group. The patients with low-to-moderate compactness-based risk who did or did not have a pathological complete response had similar OS (P=0.127)

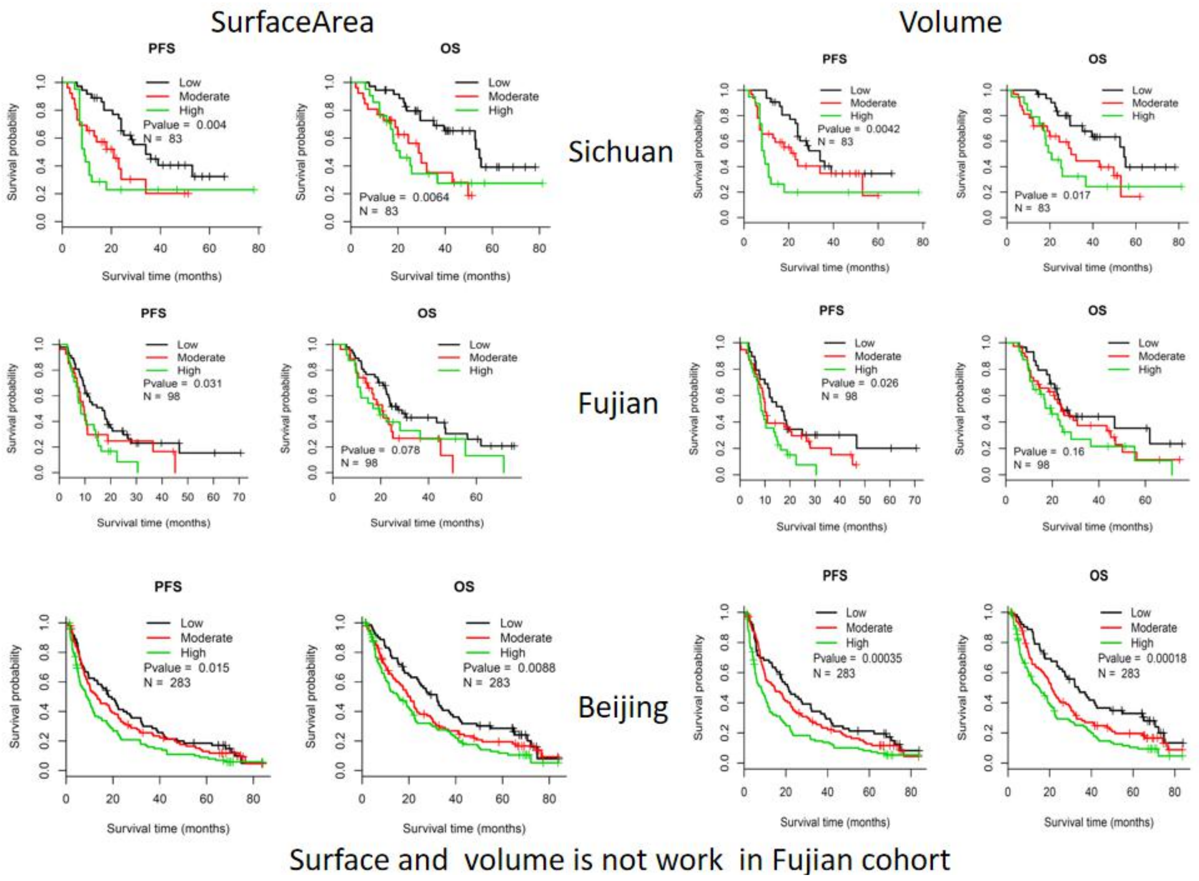

Figure S4: Validating the prognostic values of volume and surface area in two independent cohorts. We were unable to validate the associations between volume, surface, and overall survival in the Fujian dataset.

OS: overall survival, PFS: progression-free survival

Reference

1.Zhang L, et.al, Ibex: an open infrastructure software platform to facilitate collaborative work in radiomics. Med Phys 2015;42(3):1341.

2. IBEX(2015): Imaging Biomarker Explorer Software V1.0β, URL: https://aapm.onlinelibrary.wiley.com/doi/abs/10.1118/1.4908210
